# Dementia Referrals in Intellectual Disability: An Audit of Pathways and Practice in Kent and Medway MHLD Services

**DOI:** 10.1192/bjo.2026.11833

**Published:** 2026-06-30

**Authors:** Giulia Rosi, Sharna Bennett, Colin Hemmings

**Affiliations:** Kent and Medway Mental Health NHS Trust, Maidstone, United Kingdom

## Abstract

**Aims:**

Current dementia referral pathways within Mental Health of Learning Disability (MHLD) Team in Kent and Medway were analysed in order to understand existing practices while identifying opportunities for improvement. Objectives were: (1) to capture staff opinions on what MHLD should provide for people with intellectual disability (ID) and dementia, and (2) to analyse dementia-related referrals to East and West Kent MHLD over 12 months. Audit standards were drawn from NICE NG54, which advises referral of people with learning disabilities and suspected dementia to a specialist psychiatrist. We hypothesised that pathways would lack clarity.

**Methods:**

We employed a mixed-methods research design. An anonymous survey was used to collect opinions from MHLD clinicians across Kent about referral practices, assessment responsibilities, prescribing, and post-diagnostic support. We conducted a retrospective evaluation of dementia-related referrals from January-December 2024 by reviewing referral meeting documents and electronic health records. We collected information on demographics, referral sources, outcomes, and follow-up details. Descriptive statistics and thematic analysis were used.

**Results:**

Eighteen staff responded. Most (83%) supported a formalised pathway, mentioning inconsistency in the current system. Views on assessment, diagnosis, prescribing, and follow-up were divided; joint working was favoured but resource limitations were noted. Confidence gaps in diagnosis and prescribing for ID were highlighted. Twenty-seven dementia-related referrals were identified (41% East, 59% West; mean age 55.7; 63% male; 59% with Down’s Syndrome). Overall, 48% were accepted, with marked variation (82% East vs. 25% West). Psychiatric input occurred in 84% of cases, and 77% were redirected to MAS or CLDT. No confirmed diagnoses were made at initial or three-month follow-up.

**Conclusion:**

Our findings indicate differences in how dementia referrals for people with ID are managed across Kent and Medway. The staff supports the creation of a formalised pathway which would address inconsistencies and define roles among MHLD, MAS and CLDT and maintain compliance with NICE guidance. A standardised approach together with enhanced training resources could improve dementia care delivery to patients with ID.

